# Laparoscopic ultrasound eXAmination: Analysis of fellow surgeons learning curve in minimally invasive gynecologic procedures (LUXA study)

**DOI:** 10.1002/ijgo.71130

**Published:** 2026-06-10

**Authors:** Maria Teresa Giudice, Francesca Moro, Marianna Ciancia, Consuelo Russo, Diana Giannarelli, Serena Cappuccio, Matteo Loverro, Camilla Fedele, Marco D'Indinosante, Andrea Rosati, Emanuele Perrone, Francesca Ciccarone, Floriana Mascilini, Anna Fagotti, Francesco Fanfani, Antonia C. Testa

**Affiliations:** ^1^ Dipartimento Scienze della Salute della Donna e del Bambino Fondazione Policlinico Universitario Agostino Gemelli, IRCCS Rome Italy; ^2^ UniCamillus‐International Medical University of Rome Rome Italy; ^3^ Epidemiology and Biostatistics Facility, G‐STeP Generator Fondazione Policlinico Universitario A. Gemelli IRCCS Rome Italy; ^4^ Dipartimento Universitario Scienze della Vita e Sanità Pubblica Università Cattolica del Sacro Rome Italy

**Keywords:** gynecology, imaging‐guided surgery, intraoperative ultrasound, laparoscopic ultrasound, ultrasonography

## Abstract

**Objective:**

Laparoscopic ultrasound (LUS), performed with an endoscopic ultrasound probe, has shown potential in addressing challenges related to haptic perception and lesion detection in minimally invasive surgery. The aim of the present study was to evaluate the learning curve of gynecologic surgery fellows in achieving competency in LUS image acquisition during minimally invasive procedures.

**Methods:**

This prospective single‐center study included six gynecologic fellows with limited ultrasound experience who completed a one‐day structured training program. Fellows performed laparoscopic ultrasound during elective surgeries for benign conditions and early‐stage gynecologic cancers, assessing five anatomical sites. Competency was evaluated using learning curve cumulative summation (LC‐CUSUM) analysis based on image quality and examination time. Learning curve time and quality score for each surgeon and anatomical site were measured.

**Results:**

A total of 56 patients and examinations were analyzed. All fellows achieved competency within 2–5 procedures, with LC‐CUSUM showing rapid and sustained skill acquisition. Ovarian imaging required fewer attempts than myometrial wall and iliac vessel imaging, with the right external iliac vessels being the most challenging. Patient characteristics did not differ among fellows.

**Conclusion:**

After targeted training, gynecologic surgery fellows can achieve proficiency in LUS within a short learning curve. These findings support the feasibility of integrating LUS training into gynecologic surgical education and highlight its potential to enhance surgical precision and to reduce surgical overall time.

## 1 INTRODUCTION

Ultrasound examination is the primary diagnostic tool for diagnosing and managing many gynecologic conditions.^1^ In recent decades, it has played a crucial role in both benign gynecologic disorders^2^, ^3^, ^4^, ^5^ and gynecologic oncology.^6^, ^7^, ^8^ It offers an alternative to computed tomography (CT) and magnetic resonance imaging (MRI) when performed by an expert, serving as a handy and reliable imaging modality that aids in diagnosis and planning surgical management.^6^ However, preoperative imaging may not provide all the information that can only be obtained with real time intraoperative imaging (e.g., the identification of lesions not visible on the surface, the assessment of disease infiltration into surrounding organs).^9^, ^10^, ^11^, ^12^

Intraoperative ultrasound (IOUS) has become increasingly prevalent across various specialties,^13^ particularly in minimally invasive techniques (e.g., laparoscopic and robotic‐assisted procedures) where haptic feedback is limited. IOUS enables the probe to be positioned close to the structures of interest, enhancing the identification of anatomical structures, improving lesion detection when performed by an expert, and facilitating complete resection with a more precise estimation of lesion size and depth.^14^ Additionally, it provides real‐time imaging that can assist in intraoperative decision making (e.g., identification of small pelvic lesion not visible by the surgeon during laparoscopy), with the potential to enhance the precision of tailored invasive procedures and, consequently, improve surgical safety.

Specifically, the use of laparoscopic ultrasound (LUS) probe directly positioned within the abdominal cavity eliminates the abdominal or vaginal wall as a potential source of significant interference, thereby improving the visualization of pelvic and retroperitoneal lesions, enabling the detection of lesions measuring up to 3–5 mm.^15^, ^16^, ^17^ However, the use of LUS in gynecologic surgery has been limited^15^, ^16^, ^17^ and the adoption of the technique in clinical practice appears to be progressing slowly.^18^ This may be due to a lack of training in the use of laparoscopic probes among gynecologic surgeons, who are often not fully independent in the operating room and require an additional ultrasound operator during surgery. Therefore, it is crucial to train gynecologic surgeons to become proficient in LUS, enabling them to effectively use this tool in various clinical scenarios to enhance surgical accuracy, reduce complications and operating times, and ultimately improve patient care.

The objective of the present study was to evaluate the learning curve of gynecologic surgery fellows in achieving competency in the use of the laparoscopic probe.

## 2 MATERIALS AND METHODS

### 2.1 Study design

This was an industry independent prospective, single‐center feasibility study conducted at the referral oncology center, Fondazione Policlinico Universitario Agostino Gemelli IRCCS, between August 2023 and July 2024. The study period was planned to allow each fellow to perform at least six LUS examinations. Six gynecologic oncology fellows certified at level 2 of the Gynecological Endoscopic Surgical Education and Assessment (GESEA) program^19^ and with limited experience in gynecologic ultrasound (EFSUMB^20^ level 1, corresponding to 100–500 ultrasound examinations) were invited to participate in the study. The study was approved by the Local Ethics Committee (protocol no. 0017901/23, approved on June 7, 2023). Written informed consent to participate in the study was obtained from all patients.

After a dedicated training program, the fellows performed LUS on consecutive patients undergoing elective laparoscopy for early‐stage endometrial cancer, early‐stage cervical cancer and early‐stage ovarian cancer, or benign conditions, such as oncogenic mutational profile, preneoplastic endometrial pathology, benign myometrial lesions, and benign ovarian masses. Patients who had previously undergone hysterectomy or salpingo‐oophorectomy, as well as those in whom uterus and adnexa were not visible at the beginning of the laparoscopy–due to factors such as adhesions, obesity, or distorted pelvic anatomy–were excluded. We chose to assess the fellows in simpler intraoperative scenarios in order to minimize confounding factors, such as distorted anatomy and other procedural complexities. Patients were not formally randomized to individual surgeons; instead, cases were randomly assigned according to routine clinical scheduling and surgeon availability within the surgical program.

Clinical parameters of the patients included in the study were recorded, including age, body mass index (BMI, calculated as weight in kilograms divided by the square of height in meters), parity, history of previous abdominal surgery, and comorbidities. In addition, demographic and professional characteristics of the fellows were collected, including age, years as a specialist in obstetrics and gynecology, surgical field (oncology vs. non‐oncology), surgical skills (expressed according to GESEA levels^19^), and EFSUMB level.^20^

### 2.2 Procedure

The one‐day training program combined theoretical and practical instruction on LUS examination. The theoretical component included lectures and video‐recorded clinical cases focused on ultrasound assessment of pelvic anatomy. These sessions covered the use of the laparoscopic ultrasound probe, with detailed explanations of its technical features and functionality. Participants subsequently completed a hands‐on training session to practice probe manipulation and become familiar with the device.

Surgical procedures were performed in an integrated minimally invasive operating room equipped with high‐definition laparoscopic technology (Endo Alpha; Olympus). A 10‐mm flexible‐tip, high‐definition three‐dimensional video laparoscope was used. Depending on the planned procedure, either two or three 5‐mm trocars were placed in standard laparoscopic positions, as previously described.^13^ LUS examinations were performed using a 7.5‐MHz grayscale rigid and steerable laparoscopic transducer (Canon Medical Systems Europe) introduced through a 10‐mm trocar^21^ (Figure S1).

After pneumoperitoneum was established by carbon dioxide (CO_2_) insufflation, the fellow surgeon was required to evaluate five anatomical sites using LUS: the myometrial wall, the right and left ovaries, and the right and left external iliac vessels. One ultrasound image was acquired for each site, all in the sagittal plane, resulting in a total of five images per patient. These anatomical sites were selected based on their clinical relevance and their frequent assessment during gynecologic surgery. Moreover, they represent key targets for laparoscopic ultrasound, where accurate identification and characterization are essential.

The time required to evaluate each anatomical site was recorded for each fellow. A dedicated research nurse was present in the operating theater and was responsible for time measurement.

Image quality was assessed retrospectively by a single EFSUMB^20^ level 2 examiner (M.T.G.), who reviewed the stored images and did not know which fellow had acquired the images. Although only one reviewer was involved, predefined and standardized evaluation criteria were applied to minimize subjectivity and intraobserver variability. These criteria included overall image clarity, correct anatomical identification of the target structure, appropriate probe orientation in the sagittal plane, optimal adjustment of ultrasound parameters (gain, depth, and zoom), and absence of relevant artifacts. All images were reviewed using the same workstation and display settings. Each image was scored on a scale from 1 to 10, yielding a maximum total score of 50 per examination.

Fellows were not present while other fellows were performing their ultrasound scans during surgery.

A post‐procedure feedback with qualitative comments on image acquisition was provided to each fellow after all procedures by the same experienced examiner (M.T.G.) who evaluated the images.

Finally, regarding surgical procedure, the fellow gynecologic surgeons were supervised by an experienced surgeon with at least 10 years of experience in minimally invasive surgery during the surgical procedure.

### 2.3 Statistical analysis

Results are presented as absolute frequency (percentage) for nominal variables and as mean (standard deviation [SD]) for continuous variables. The statistical significance of differences among patients and surgeons was assessed using the Chi‐square test for categorical variables and analysis of variance (ANOVA) test for continuous variables. *P* values less than 0.05 were considered statistically significant.

The statistical methods were applied after verifying the normality assumption for the variables using the Shapiro–Wilk test.

Learning curve‐cumulative summation (LC‐CUSUM) analysis was conducted to evaluate each fellow's performance based on the time required to complete the entire examination (i.e., the assessment of 5 anatomical sites) and on quality score. Competency was defined a priori based on a composite criterion including both technical performance and efficiency by an EFSUMB level 3 (ACT). Specifically, a fellow was considered competent when achieving a quality score > 5 in at least three of the five anatomical sites and completing the examination within 120 s.

The LC‐CUSUM charts consists in determining when the fellow performance is sufficiently far away from an unsatisfactory performance level to consider the performance as acceptable and it supposes that a newcomer fellow is not performing adequately at the start of monitoring.

The null hypothesis was set at a failure rate of 30% (unacceptable failure rate), while the alternative hypothesis was set at a failure rate of 10% (acceptable failure rate).

The type 1 and 2 errors of 0.10 (probability of claiming that the trainee is competent while they are not) and 0.10 (probability of rejecting the fact that the trainee has reached their competency), respectively, entailed a limit of *h* = 1.6.

All statistical analyses were performed with R software (version 4.2.1, R Core Team 2020, R: A language and environment for statistical computing; R Foundation for Statistical Computing, Vienna, Austria; https://www.R‐project.org/).

All clinical and ultrasound data were retrieved prospectively from patient records using a data collection system (Research Electronic Data Capture [REDCap] software; https://projectredcap.org/) and then entered into an Excel file (Microsoft Office Excel 2011 Redmond, WA, USA) by the principal investigator, which was used for descriptive statistical analysis.

## 3 RESULTS

Six fellow surgeons participated and all were certified at level 2 of the GESEA program.^19^ The professional characteristics of fellows are summarized in Table S1. A total of 56 patients were recruited and included in the analysis. One fellow performed 13 LUS examinations, another 12 examinations, one performed nine, two performed eight, and one performed six.

Demographic and clinical characteristics of the study population, both overall and stratified by each fellow, are summarized in Table 1. The mean age of the population was 51.23 (±10.62) years, and the mean BMI was 24.16 (±3.81). Previous abdominal surgery was reported in 36/56 (64.3%) patients. No statistically significant differences in patient characteristics were observed among the six fellows. Regarding the surgical indications, 44/56 (78.6%) patients underwent surgery for early stage gynecologic cancers whereas 12/56 (21.4%) patients underwent surgery for benign indications (e.g., prophylactic procedures, atypical endometrial hyperplasia, benign adnexal masses).

**TABLE 1:** Patient demographics and clinical characteristics.

Patient data	Overall *n* = 56	Fellow 1 *n* = 8	Fellow 2 *n* = 12	Fellow 3 *n* = 8	Fellow 4 *n* = 9	Fellow 5 *n* = 6	Fellow 6 *n* = 13	*P*
Age, years; mean (SD)	51.23 (10.62)	50.0 (7.79)	51.4 (8.74)	54.2 (14.6)	47 (11.01)	54.8 (11.6)	51.31 (11.01)	0.733
Height, cm; mean (SD)	163.1 (5.56)	163.4 (5.87)	164.3 (4.33)	162.0 (6.34)	161.7 (5.45)	160.2 (5.45)	164.8 (4.94)	0.499
Weight, kg; mean (SD)	64.28 (10.5)	67.12 (11.01)	64.79 (11.22)	67.5 (12.58)	56.89 (4.07)	62.17 (8.08)	66.15 (11.6)	0.27
BMI mean (SD)	24.16 (3.81)	25.09 (3.51)	23.98 (4.00)	25.73 (4.78)	21.89 (2.73)	24.30 (3.79)	24.3 (3.76)	0.417
BMI classification								0.342
Underweight *n* (%)	2 (3.57)	‐	‐	‐	2 (22.22)	‐	‐	
Normal weight *n* (%)	35 (62.5)	4 (50)	8 (66.67)	6 (75)	6 (66.6)	4 (66.6)	7 (53.8)	
Overweight *n* (%)	14 (25.0)	3 (37.5)	2 (16.67)	1 (12.5)	1 (11.1)	2 (33.3)	5 (38.4)	
Obesity *n* (%)	5 (8.92)	1 (12.5)	2 (16.67)	1 (12.5)	‐	‐	1 (7.69)	
Parity, *n* (%)	45 (80.35)	8 (100)	11 (91.67)	5 (62.5)	7 (77.78)	5 (83.33)	9 (69.23)	0.343
Previous abdominal surgery, *n* (%)	36 (64.3)	5 (62.5)	9 (75)	5 (62.5)	6 (66.6)	2 (33.3)	9 (69.2)	0.655
Comorbidities, *n* (%)	26 (46.4)	4 (50)	5 (41.67)	5 (62.5)	5 (55.5)	2 (33.3)	5 (38.4)	0.897

*Note*: BMI, calculated as weight in kilograms divided by the square of height in meters.

Abbreviations: BMI, body mass index; SD, standard deviation.

The mean time required to evaluate each anatomical site for each fellow across all procedures, as well as the mean image quality scores, are presented in Table 2. For each anatomical site, the ANOVA test revealed no statistically significant differences in the mean time required to obtain images among the fellows. The mean quality scores differed among fellows with regard to myometrial wall image, right and left ovary images. The LC‐CUSUMs showing the competency of each fellow for the entire examination are shown in Figure 1.

**TABLE 2:** Mean time and quality score for each surgeon and for each anatomical site.

Time (seconds)	Fellow 1 Mean (SD)	Fellow 2 Mean (SD)	Fellow 3 Mean (SD)	Fellow 4 Mean (SD)	Fellow 5 Mean (SD)	Fellow 6 Mean (SD)	*P*
Entire examination	117.6 (56.7)	159.4 (100.3)	128.0 (71.7)	133.6 (65.9)	127.3 (56.9)	128.8 (92.1)	0.712
Right ovary image	18.62 (8.36)	20.42 (14.1)	20.00 (17.3)	22.78 (16.9)	20.33 (6.8)	20.92 (15.5)	0.979
Left ovary image	19.00 (7.80)	26.00 (22.4)	18.62 (10.8)	19 (19.2)	24.50 (10.04)	20.92 (13.17)	0.756
Right iliac vessels image	33.25 (27.2)	40.25 (44.2)	36.50 (26.8)	46 (26.3)	29.5 (23.3)	33.54 (32.9)	0.685
Left iliac vessels image	24.75 (11.52)	50.42 (41.8)	35.88 (32.7)	30.78 (37.3)	32.0 (26.9)	35.15 (44.16)	0.151
Myometrial wall image	22.0 (17.14)	22.33 (16.7)	17.00 (7.83)	15.00 (15.2)	21.00 (3.26)	18.23 (15.51)	0.809
**Quality score**							
Right ovary image	6.5 (0.7)	6.9 (0.45)	6.0 (0.53)	6.1 (0.78)	5.3 (1.50)	6.8 (1.14)	0.013
Left ovary image	7 (0.9)	6.6 (0.51)	6 (0.75)	6.2 (0.83)	5.5 (1.30)	6.5 (0.96)	0.041
Right iliac vessels image	6.2 (0.8)	6.6 (1.31)	6.0 (0.75)	5.6 (0.72)	5.5 (1.37)	6 (0.75)	0.254
Left iliac vessels image	6.8 (0.64)	6.4 (1.24)	5.9 (0.83)	5.9 (0.33)	5.7 (1.87)	6 (0.71)	0.187
Myometrial wall image	6.3 (1.06)	6.9 (0.99)	5.9 (0.83)	6.1 (0.33)	6.0 (1.26)	7.0 (0.91)	0.031

Abbreviation: SD, standard deviation.

**FIGURE 1:**
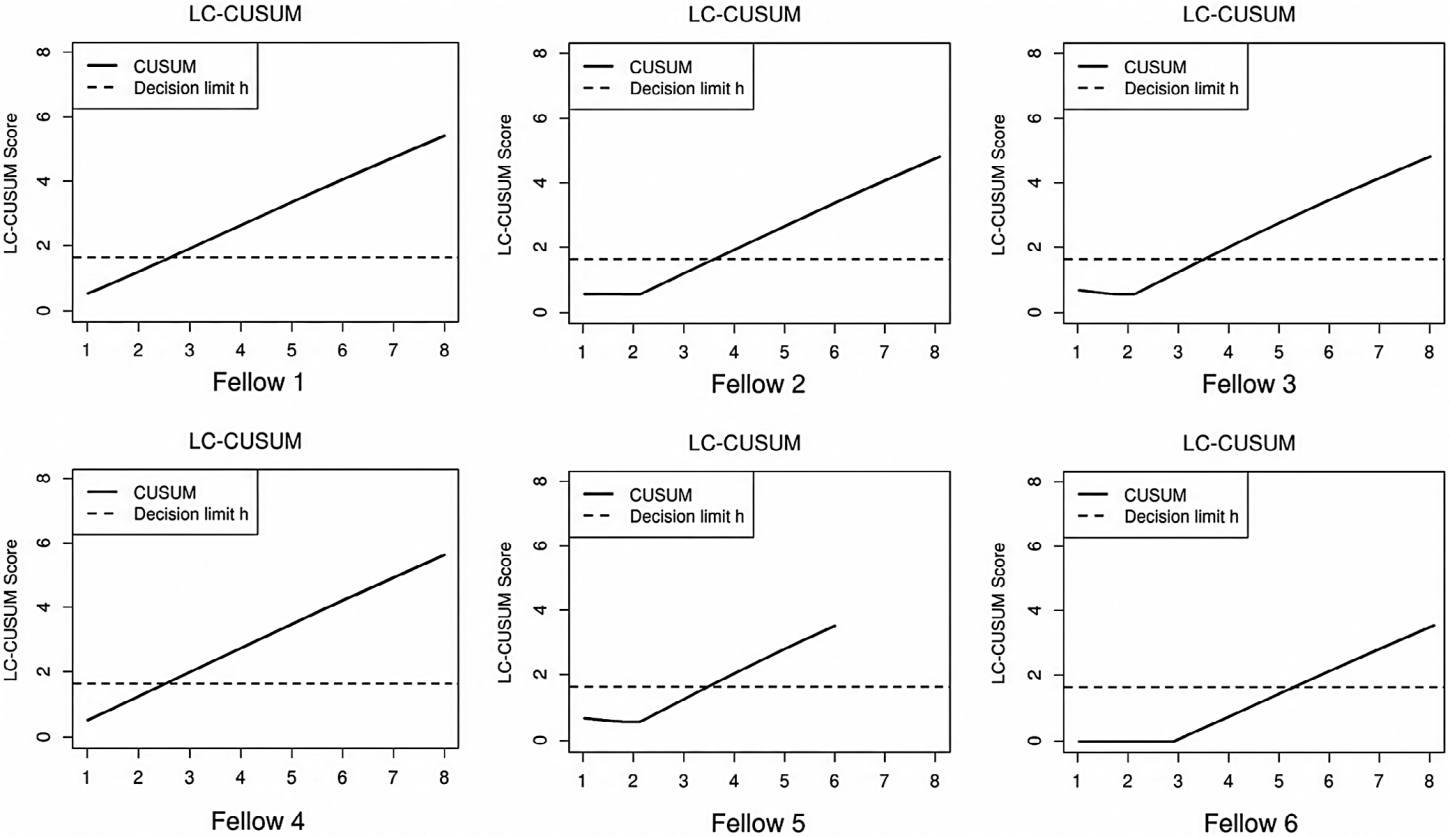
Learning cumulative summation curve LC‐CUSUM) results for the six fellows. The dashed line represents the limits *h* or “line of competence.” For as long as the LC‐CUSUM score remains below the decision limit (*h* = 1.6) the performance of the trainee is considered as inadequate.

Fellows 1 and 4 demonstrated competencies after two examinations and maintained acceptable performance throughout all subsequent procedures. Fellows 2, 3 and 5 required three procedures to achieve proficiency, after which they also maintained a positive performance trajectory. Finally, fellow 6 required five examinations to reach proficiency, after which he remained proficient in the subsequent assessments. Therefore, all fellows demonstrated steady and reliable competence in performing the procedures with the LUS probe, maintaining an adequate skill level throughout the observation period.

The LC‐CUSUMs showing the competency of each fellow for each anatomical site are shown in Figures S2–S6. By analyzing these curves, generated for each fellow across the images of the five different anatomical sites, we observed that fewer attempts were required to achieve competency for adequate image acquisition of the ovary, either right or left, compared with the myometrial wall and the iliac vessels. Overall, the most challenging image for our fellows to acquire with good quality was that of the right external iliac vessels.

## 4 DISCUSSION

This prospective study demonstrated that fellows with limited previous ultrasound experience were able to acquire adequate LUS images after a short structured training program. Competency was achieved after only a limited number of examinations, suggesting that the acquisition of basic LUS skills may be feasible within a relatively short training period.

The LC‐CUSUM analysis showed differences in the learning process according to the anatomical site evaluated. Ovarian imaging required fewer attempts to achieve proficiency, probably because of the more recognizable morphology and relatively consistent ultrasound appearance of the ovaries. In contrast, assessment of the myometrial wall and external iliac vessels required a longer learning curve, likely reflecting the greater technical complexity associated with probe positioning, spatial orientation, and image optimization during minimally invasive surgery.

These findings are clinically relevant because the use of IOUS during minimally invasive gynecologic surgery is progressively increasing.^4^, ^10^, ^11^, ^12^, ^14^, ^21^, ^22^, ^23^, ^24^, ^25^ Although several studies have investigated the diagnostic role of LUS, limited evidence is available regarding the training process required for surgeons to achieve competency in this technique.

Previous investigations mainly focused on the clinical applications of LUS. Levine et al.^16^ reported that LUS was more sensitive than preoperative imaging for the detection of small uterine fibroids. Similarly, Patel et al.^24^ demonstrated that LUS identified additional fibroids not detected during preoperative evaluation. Puppo et al.^25^ also described the usefulness of LUS in the assessment of bowel endometriosis infiltration. However, these studies did not evaluate the surgeons' learning curve, the level of ultrasound expertise required, or the time needed to perform intraoperative ultrasound assessment. Therefore, comparison with our findings remains limited.

The present study suggests that a standardized training pathway may allow gynecologic surgeons to independently perform basic LUS examinations during surgery. This could have practical implications in the operating room, particularly in minimally invasive procedures where the availability of an additional ultrasound operator may be limited. Autonomous use of LUS by the surgeon may improve intraoperative workflow and facilitate identification of anatomical structures and target lesions.

This study had several strengths. The prospective design allowed standardized evaluation of the learning process, and all fellows had similar surgical and ultrasound experience, reducing heterogeneity among participants. Nevertheless, some limitations should be acknowledged.

First, the number of included cases was relatively small, which may have affected the robustness of the LC‐CUSUM analysis. Second, patients with advanced gynecologic malignancy or complex pelvic disease were excluded. As a result, the study population consisted mainly of patients with relatively uncomplicated anatomy, which may have facilitated image acquisition and contributed to the rapid achievement of competency observed among fellows. Another limitation is that image quality assessment was performed by a single expert examiner. Although predefined evaluation criteria and retrospective assessment by an examiner who did not know which fellow had acquired each image were used to improve consistency, interobserver variability could not be assessed. Furthermore, the inclusion of fellows with similar professional backgrounds may limit the generalizability of the findings to surgeons with different levels of expertise or different training settings.

Future studies including larger populations and more complex surgical scenarios are needed to better define the applicability of LUS training in routine gynecologic surgery and to validate these preliminary findings.

## 5 CONCLUSION

In the era of personalized and technology‐assisted medicine, a non‐invasive tool as IOUS can be an extraordinary instrument for tailoring surgical procedures, especially in more complex conditions, and should be integrated into routine surgical practice once adequate training is provided.

It is worth investing in training the next generation of surgeons with expertise in both surgery and ultrasound in order to increase the number of ultrasound‐guided surgical procedures.

This will enable them to discover new applications and develop innovative, alternative devices.

In conclusion, our results suggest that surgeon fellows can successfully perform LUS, with their skills improving with each examination. However, the extent to which this technique enhances surgical precision, decision making, and surgical outcomes remains unclear and should be further investigated in other studies and settings.

## AUTHOR CONTRIBUTIONS

MTG, FMoro and MC: Had full access to all the data in the study and take responsibility for the integrity of the data and the accuracy of the data analysis. FMoro, FMascilini, FF and ACT: Concept. FMoro: Design. MTG, FMoro, MC, CR, FC, FMascilini and ACT: Data collection or processing. MTG, FMoro, MC and AC: Analysis or interpretation. MTG and MC: Literature search. SC, ML, CF, MD'I, AR, EP, GS, AF and FF: Surgical and medical practices. MTG, FMoro and ACT: Writing. ACT: Supervision. All authors accept responsibility for the paper as published.

## FUNDING INFORMATION

This research did not receive any specific grant from funding agencies in the public, commercial, or not‐for‐profit sectors.

## CONFLICT OF INTEREST STATEMENT

All authors have completed the ICMJE uniform disclosure form at www.icmje.org/disclosure‐of‐interest/and declare they have nothing to disclose.

## ETHICS STATEMENT

The present study was approved by the referral regional ethical committee (Prot. N. 0017901/23, approved on 7 June 2023).

## Supporting information


**Figure S1:** Laparoscopic ultrasound equipment. (A) Laparoscopic ultrasound probe (B) Laparoscopic ultrasound probe (white asterisk) covered with steril envelope and inserted through a 10 mm trocar.
**Figure S2:** LC‐CUSUM (Learning Cumulative Summation Curve) results for the 6 fellows with reference to the left external iliac vessels. The dashed line represents the limits *h* or “line of competence.” For as long as the LC‐CUSUM score remains below the decision limit (*h* = 1.6) the performance of the trainee is considered as inadequate.
**Figure S3:** LC‐CUSUM (Learning Cumulative Summation Curve) results for the 6 fellows with reference to the right external iliac vessels. The dashed line represents the limits *h* or “line of competence.” For as long as the LC‐CUSUM score remains below the decision limit (*h* = 1.6) the performance of the trainee is considered as inadequate.
**Figure S4:** LC‐CUSUM (Learning Cumulative Summation Curve) results for the 6 fellows with reference to the right ovary. The dashed line represents the limits *h* or “line of competence.” For as long as the LC‐CUSUM score remains below the decision limit (*h* = 1.6) the performance of the trainee is considered as inadequate.
**Figure S5:** LC‐CUSUM (Learning Cumulative Summation Curve) results for the 6 fellows with reference to the left ovary. The dashed line represents the limits *h* or “line of competence.” For as long as the LC‐CUSUM score remains below the decision limit (*h* = 1.6) the performance of the trainee is considered as inadequate.
**Figure S6:** LC‐CUSUM (Learning Cumulative Summation Curve) results for the 6 fellows with reference to the myometrial wall. The dashed line represents the limits *h* or “line of competence.” For as long as the LC‐CUSUM score remains below the decision limit (*h* = 1.6) the performance of the trainee is considered as inadequate.
**Table S1:** Surgeons' professional characteristics.

## Data Availability

Additional data and related information are available from the corresponding author upon request.
