# Supplementary material for: Laparoscopic ultrasound eXAmination: Analysis of fellow surgeons learning curve in minimally invasive gynecologic procedures (LUXA study)
Source: Int J Gynaecol Obstet. 2026 Jun 10;175(2):969–76. doi: 10.1002/ijgo.71130 (PMC13674825; doi:10.1002/ijgo.71130)
Supplement: Supplementary file 1 [file IJGO-175-969-s001.docx]

**Supplementary material**

**Table S1.** Surgeons’ professional characteristics.

| **Surgeon data** | **Surgeon 1** | **Surgeon 2** | **Surgeon 3** | **Surgeon 4** | **Surgeon 5** | **Surgeon 6** |
| --- | --- | --- | --- | --- | --- | --- |
| **Examination performed,** n | 8 | 12 | 8 | 9 | 6 | 13 |
| **Age,** years | 34 | 31 | 34 | 33 | 35 | 36 |
| **Graduate,** years (%)  2  3  4  5 | -  2 (25)  6 (75)  - | 12 (100)  -  -  - | -  8 (100)  -  - | -  9 (100)  -  - | -  -  -  6 (100) | -  -  1 (7.69)  12 (92.3) |
| **Surgical field**, n (%)  Benign pathology  Oncology | 7 (87.5)  1 (12.5) | -  12 (100) | -  8 (100) | 4 (44.5)  5 (55.5) | -  6 (100) | 1 (7.69)  12 (92.3) |
| **Surgical skills (GESEA levels)**, n (%)  Level 1  Level 2 | -  8(100) | -  12 (100) | -  8 (100) | -  9 (100) | -  6 (100) | -  13 (100) |
| **Ultrasound skills (EFSUMB levels)**, n (%)  Level 1  Level 2  Level 3 | 8 (100)  -  - | 12 (100)  -  - | 8 (100)  -  - | 9 (100)  -  - | 6 (100)  -  - | 13 (100)  -  - |

Note: GESEA= Gynecological Endoscopic Surgical Education and Assessment; EFSUMB= European Federation of Societies for Ultrasound in Medicine and Biology.

**Figure S1.** Laparoscopic ultrasound equipment.

A) Laparoscopic ultrasound probe B) Laparoscopic ultrasound probe (white asterisk) covered with steril envelope and inserted through a 10 mm trocar.

**Figure S2.** LC-CUSUM (Learning Cumulative Summation Curve) results for the 6 fellows with reference to the left external iliac vessels.

The dashed line represents the limits h or ‘‘line of competence.’’ For as long as the LC-CUSUM score remains below the decision limit (h=1.6) the performance of the trainee is considered as inadequate.

**Figure S3.** LC-CUSUM (Learning Cumulative Summation Curve) results for the 6 fellows with reference to the right external iliac vessels.

The dashed line represents the limits h or ‘‘line of competence.’’ For as long as the LC-CUSUM score remains below the decision limit (h=1.6) the performance of the trainee is considered as inadequate.

**Figure S4.** LC-CUSUM (Learning Cumulative Summation Curve) results for the 6 fellows with reference to the right ovary.

The dashed line represents the limits h or ‘‘line of competence.’’ For as long as the LC-CUSUM score remains below the decision limit (h=1.6) the performance of the trainee is considered as inadequate.

**Figure S5.** LC-CUSUM (Learning Cumulative Summation Curve) results for the 6 fellows with reference to the left ovary.

The dashed line represents the limits h or ‘‘line of competence.’’ For as long as the LC-CUSUM score remains below the decision limit (h=1.6) the performance of the trainee is considered as inadequate.

**Figure S6.** LC-CUSUM (Learning Cumulative Summation Curve) results for the 6 fellows with reference to the myometrial wall

The dashed line represents the limits h or ‘‘line of competence.’’ For as long as the LC-CUSUM score remains below the decision limit (h=1.6) the performance of the trainee is considered as inadequate.
